# Trends and patterns in hand injuries during the a pandemic

**DOI:** 10.1016/j.jorep.2026.100917

**Published:** 2026-08

**Authors:** Thijs H. Geerdink, Marcel G.W. Dijkgraaf, Ruben N. van Veen, Dorien A. Salentijn

**Affiliations:** a Department of Trauma Surgery, OLVG, Jan Tooropstraat 164, 1061 AE, Amsterdam, the Netherlands; b Department of Epidemiology and Data Science, Amsterdam UMC - University of Amsterdam, Meibergdreef 9, 1105 AZ, Amsterdam, the Netherlands; c Department of Radiology and Nuclear Medicine, Amsterdam UMC - University of Amsterdam, Meibergdreef 9, 1105 AZ, Amsterdam, the Netherlands

**Keywords:** Hand injury, Health policy, Emergency medicine, Pandemic, Surgery, Management

## Abstract

**Purpose:**

A significant portion of the musculoskeletal extremity injuries are hand and finger injuries. Strict government measures during the COVID-19 pandemic led to changes in incidence, types and mechanisms of hand injuries. Data is needed to develop targeted strategies for management and treatment of these injuries effectively in case of future pandemics.

**Methods:**

In this retrospective study, data were gathered during calendar weeks 11-18 in the years 2019 (pre-pandemic) and 2020 (pandemic). Patients were categorized into adults and children (<18 years). Type of hand injury was further categorized into: carpal injuries or dislocations, metacarpal injuries or dislocations, phalangeal fractures, finger dislocations and mallet fingers.

**Results:**

During the pandemic the absolute number of hand injuries decreased by 55%, the decrease was larger in children (73%) then in adults (48%). In children, sports as underlying trauma mechanism decreased from 50% to 16%, metacarpal injuries due to physical interactions doubled proportionally from 33% to 70%. In adults, hand injuries caused by sports decreased while injuries resulting from physical interactions increased from 9% to 20%, more patients were operated on (13% versus 7%; *p* = 0.018). Metacarpal injuries proportionally increased and experienced a higher operation rate (20% versus 7%; *p* = 0.026), mallet patients were more often female (50% versus 13%; *p* = 0.031).

**Conclusions:**

Restrictions during a pandemic lead to an absolute decrease in number of patients with hand and finger injuries, cause a shift in underlying trauma mechanisms from sport to physical interactions, result in a significantly higher probability of needing operative treatment when being an adult patient.

**Level of evidence:**

IV

## 1 Introduction

A significant portion of musculoskeletal extremity injuries observed in emergency departments are hand and finger injuries^1^, ^2^, ^3^. These injuries are often associated with sports activities 4, 5, 6, 7, 8. The strict government COVID-19 measures, including lockdowns, led to changes in the incidence, types and mechanisms of hand injuries 9, 10, 11, 12.

The aim of the current study was to assess the influence of the pandemic and its concomitant restrictions on the incidence of different types of hand injuries, as well as on patient characteristics, underlying trauma mechanisms and treatment approach (i.e. non-operative versus operative treatment). This information might be helpful to develop targeted strategies for management and treatment of these injuries effectively in case of future pandemics or government restrictions including lockdowns or curfews.

## 2 Methods

### 2.1 Study population and data collection

A retrospective study was carried out in an urban level-2 trauma center and teaching hospital in the Netherlands. All consecutive patients with hand injuries were included if they presented at the Emergency Departments (ED) during calendar weeks 11-18 in the years 2019 (pre-pandemic year) and 2020 (pandemic year). Patients were categorized into two groups: adults (≥18 years) and children (<18 years).

Ethical approval for this study was obtained by the institutional review board of our hospital (WO 22.129). Informed consent was not sought for the present study because data was anonymized.

Electronic patient records were used to gather data regarding gender, age, type of injury (classified according to the ICD-10 code), treatment approach (non-operative versus operative), and trauma mechanism. Type of hand injury was further categorized into: carpal fractures or dislocations, metacarpal fractures or dislocations, phalangeal fractures, finger dislocations and mallet fingers. The trauma mechanisms leading to these injuries were categorized into three main groups for both children and adults: 1) sports-related, 2) everyday activities, and 3) injuries resulting from physical interactions. These categories were further subdivided for children and adults separately.

For children, sport-related activities were subdivided into: soccer; basketball; gymnastics/or rugby/or boxing/or dancing; skating/or stepping; field hockey. Everyday activities were subdivided into: injury caused by object (e.g. finger between door); playing in-/outside/or fall from bicycle. Injuries resulting from physical interactions were subdivided into: hit against the wall; friendly fight; unfriendly fight.

For adults, sport-related activities were subdivided into: low-speed individual sports (e.g. swimming, yoga, weightlifting, (table)tennis, boxing); high-speed individual sports (e.g. cycling, ice-skating, skiing, skateboarding, athletics); soccer/or American football/or rugby; field/or ice-hockey; basketball/or volleyball/or handball). Daily activities were subdivided into: injury caused by object; fall outside (tripped/fall with a two-wheeler); fall inside (tripped/or during domestic) Injuries resulting from physical interactions were subdivided into: hit against the wall; friendly fight; unfriendly fight.

### 2.2 Outcomes

The primary outcome was differences in the incidence of patients with hand and finger injuries presenting at the ED in the years 2019 (pre-pandemic) and 2020 (pandemic).

Secondary outcomes were the differences in types of hand injuries (analyzed using stocked bar charts), the differences in trauma mechanisms (analyzed using stocked bar charts), and the differences in treatment approach (non-operative versus operative) for both children and adults. Tertiary outcomes were between-group baseline differences for operatively versus non-operatively treated patients, as well as for the specific types of hand injuries.

### 2.3 Statistical analysis

Baseline characteristics were reported using descriptive statistics. The Mann Whitney-U test or Chi-squared tests/Fisher's exact tests were used as appropriate for the assessment of statistical differences in gender and age. A *p*-value <0.05 indicated statistical difference. All analyses were performed using SPSS version 29.0.^13^

## 3 Results

In calendar weeks 11-18 in the year 2019 (pre-pandemic), a total of 442 patients with hand and finger injuries presented to our ED, including 323 adults and 119 children. During the same weeks in the year 2020 (pandemic), a total of 201 patients with hand and finger injuries presented to our ED, including 169 adults and 32 children. No differences were found in age or gender (Table 1). The absolute reduction of patients who presented to the ED comparing pre-pandemic year 2019 and pandemic year 2020 was 241 (55%). For children, this was 87 (73%) and for adults 154 (48%).

**Table 1:** Patient characteristics.

**Characteristics**	Children			Adults		
	**2019** n = 119	**2020** n = 32	**P-value**	**2019** n = 323	**2020** n = 169	**P-value**
Age: median (IQR^a^)	11 (7-14)	12 (9-15)	0.37	36 (26-53)	32 (26-50)	0.21
Female: n (%)	50 (42)	9 (28)	0.11^b^	116 (36)	65 (39)	0.58^b^
Operated n (%)	4 (3)	1 (3)	0.71^b^	22 (7)	22 (13)	**0.02**^b^
*Age: median (IQR)*	*5 (2-11)*	*16 (16-16)*	*0.16*	*34 (26-49)*	*33 (28-55)*	*0.45*
*Female: n (%)*	*2 (50)*	*0 (0)*	*0.60*	*4 (18)*	*8 (36)*	*0.16*

a IQR: Interquartile range.

b Fischer exact.

### 3.1 Children

#### 3.1.1 Type of injury

**Fig. 1:**
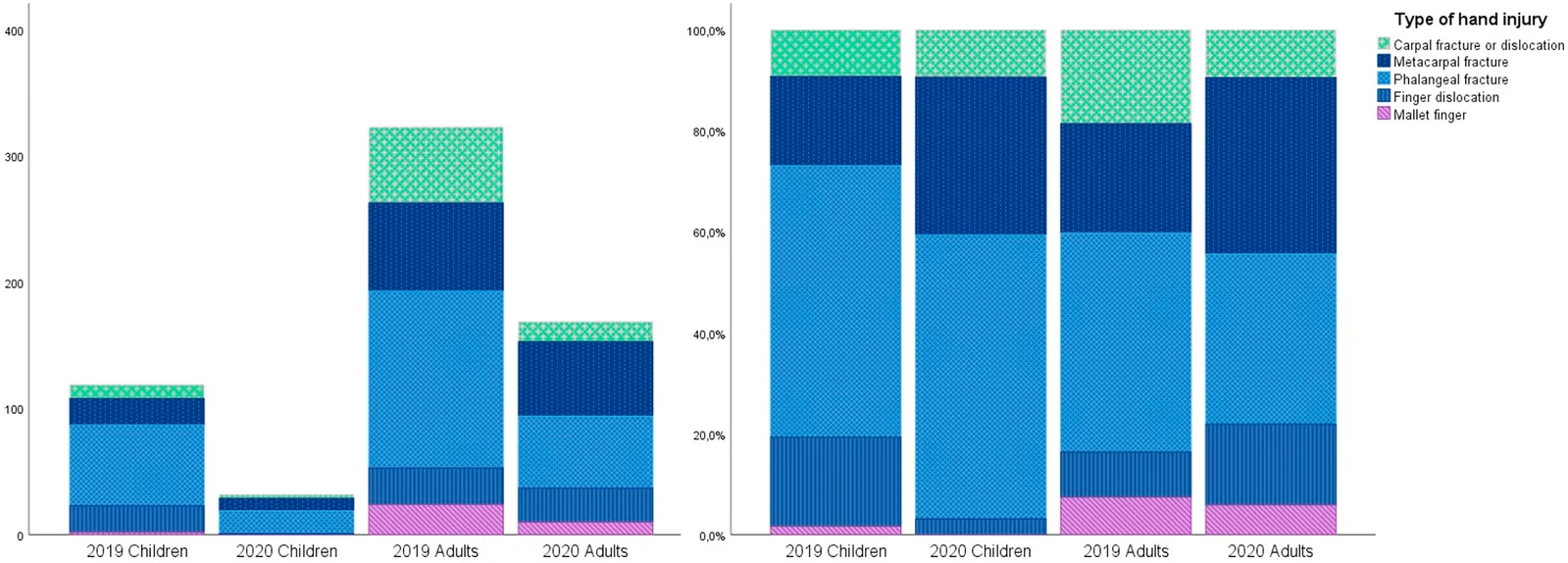
Type of hand injury per year in children and adults, value scaled (left) scaled 100% (right).

The following reductions were found, ranging from greatest reduction to smallest reduction (pre-pandemic versus pandemic): phalangeal fractures (64 versus 18 patients), finger dislocations (21 versus 1), metacarpal injuries (21 versus 10), carpal injuries (11 versus 3) and mallet fingers (2 versus 0). Proportionally, finger dislocations reduced most (18% versus 3% of all hand injuries) while phalangeal fractures slightly increased (54% versus 56%). Metacarpal injuries almost doubled proportionally (18% versus 31%) and carpal injuries occurred similarly at 9% (Fig. 1).

#### 3.1.2 Trauma mechanisms

**Fig. 2:**
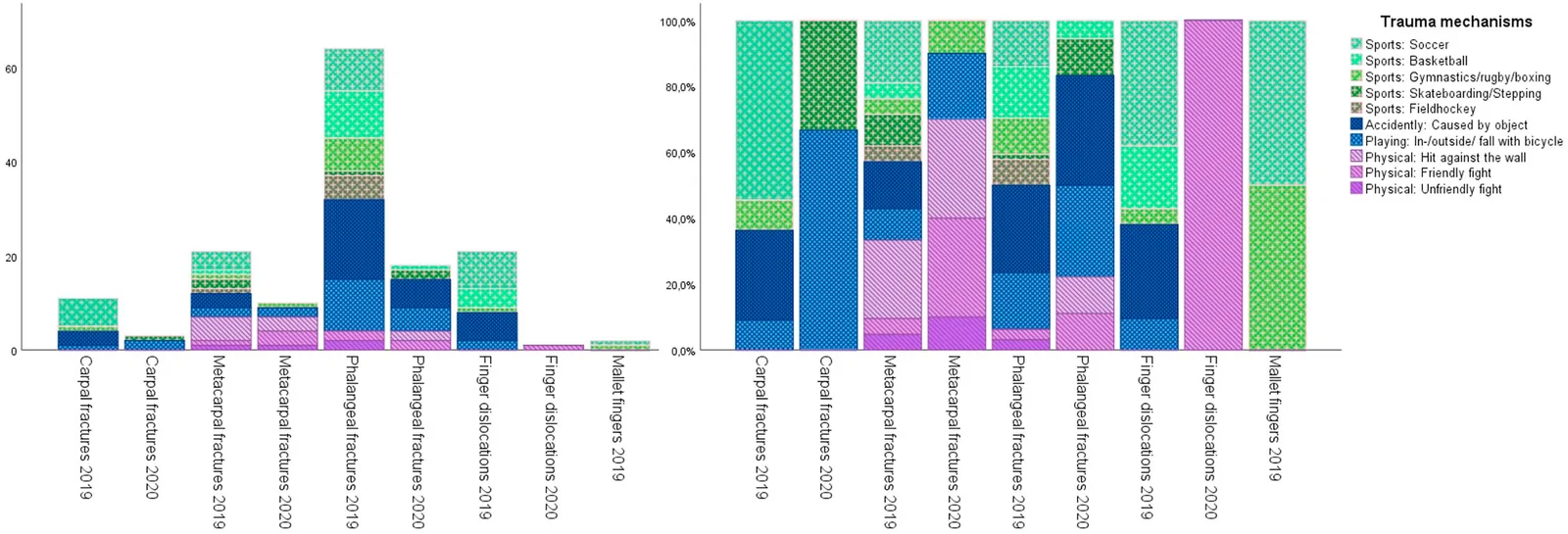
Underlying trauma mechanisms per type of injury per year in children.

Most notably were changes in organized sports, i.e. less frequently leading to hand and finger injuries, including soccer (24% versus 0%), basketball (13% versus 3), field hockey (5% versus 0%), and gymnastics/rugby/boxing (9% versus 3%). However, there was an increase in injuries caused by individual non-organized sports such as skating/stepping (3% versus 9%) and by playing in-/outside/bicycling (13% versus 28%). Injuries resulting from physical interactions increased for hit against the wall (4% versus 16%) and friendly fights (3% versus 19%). Injuries due to unfriendly fights remained stable at 3% (Fig. 2 and Table S1).

Overall, the contribution of sports leading up to hand and finger injuries decreased from 50% (pre-pandemic) to 16% (pandemic). Carpal injuries were more frequently a result of playing in-/outside/bicycling, and phalangeal fractures of playing in-/outside/bicycling in combination with physical interactions. Finger dislocations were more often physical-related injuries. Mallet fingers were non-existent during the pandemic. Metacarpal injuries due to physical interactions doubled proportionally from 33% to 70% (Fig. 2 and Table S1).

### 3.2 Adults

#### 3.2.1 Types of injury

The following reductions in number of patients were found, ranging from greatest reduction to smallest reduction (pre-pandemic versus pandemic): phalangeal fractures (140 versus 57), carpal injuries (60 versus 16), mallet fingers (24 versus 10), metacarpal injuries (70 versus 59) and finger dislocations (29 versus 27). Proportionally, carpal injuries halved (19% versus 10%), phalangeal fractures reduced from 43% to 34%, mallet finger injuries reduced from 7% to 6% and finger dislocations almost doubled (9% to 16%). Metacarpal injuries increased from 22% to 35% (Fig. 1).

#### 3.2.2 Trauma mechanisms

During the pandemic, hand and finger injuries were less frequently caused by; sports (27% versus 20%), tripping inside/domestic work (15% versus 14%), objects (16% to 15%). Injuries resulting from physical interactions or falling outside increased respectively from 9% to 20 and from 28% to 32%.

**Fig. 3:**
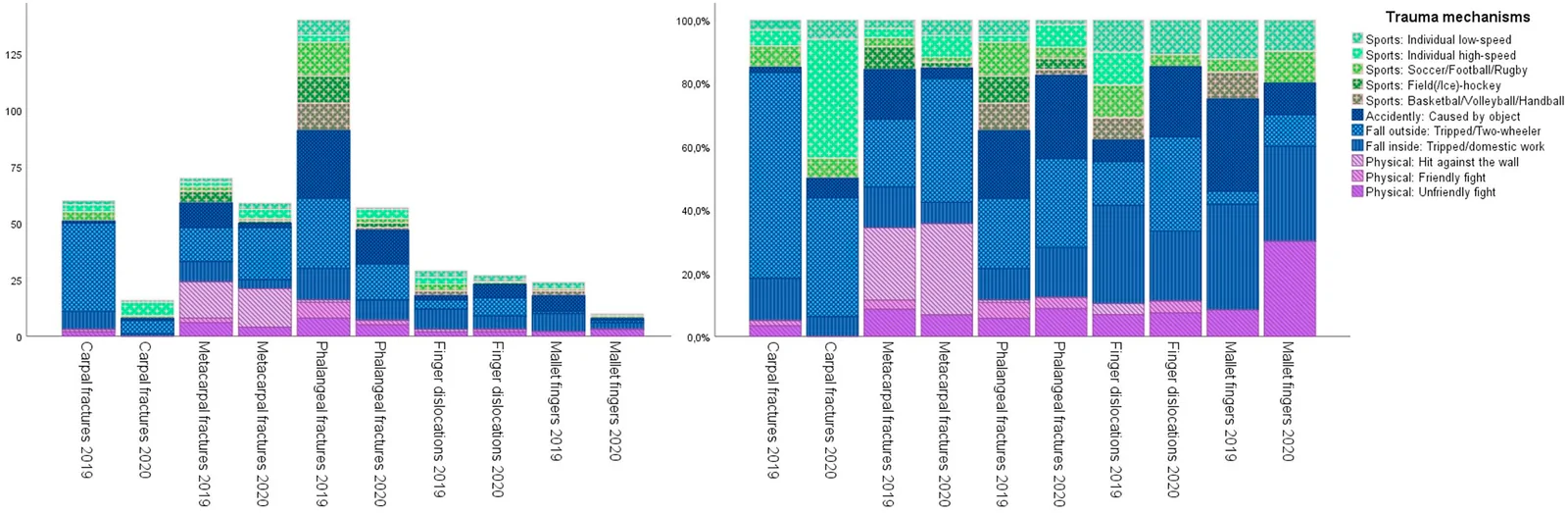
Underlying trauma mechanisms per type of injury per year in adults.

Injuries occurred during organized sports proportionally decreased (field-/ice-hockey 5% versus 2%; soccer/football/rugby 8% versus 4%; basketball/or volleyball/or handball 5% versus 1%), while injuries occurred during individual high-speed sports proportionally increased from 3% to 8%. Individual low-speed sports remained equal around 5%. When analyzing trauma mechanisms per specific injury, there were several notable changes. Carpal injuries were more frequently caused by individual high speed sports (5% versus 38%) and metacarpal injuries were much more often caused by tripping outside/fall with two-wheeler (21% versus 39%). For all other changes, see Fig. 3 and Table S2.

### 3.3 Treatment approach

Children were not operated on more often during the pandemic where in adult patients a significant increase in operation rate was found (7% versus 13%; *p* = 0.02).

When looking at the operatively treated patients specifically, no significant differences were found in age or gender for both children and adults comparing pre-pandemic year 2019 with pandemic year 2020 (Table 1).

### 3.4 Patient characteristics per type of hand injury

When looking separately at the five types of hand injuries a significantly higher operation rate was found in adult patients with metacarpal injuries during the pandemic year (20% versus 7%; *p* = 0.026), and a significantly higher percentage of mallet finger patients were female. The age of patients presenting with mallet fingers during the pandemic lowered from 49 to 32 years old. The operation rate for adult phalangeal fractures increased from 1% to 9%. No other significant differences were found for both children and adults per injury type e.g. concerning age, gender or operation rate (Table 2).

**Table 2:** Differences in age, gender and operative vs non-operative treatment per type of hand injury per year in children and adults.

***Children n = 151***	Age			Gender (F)			Operated		
	2019	2020	P-value	2019	2020	p-value	2019	2020	p-value
	*median; [IQR]*			*n (%)*			*n (%)*		
Carpal fractures	14 [11-16]	12 [9−]	0.31	3 (27)	1 (33)	0.67*	0 (0)	0 (0)	-
Metacarpal fractures	14 [9-15]	14 [13-16]	0.40	5 (24)	1 (10)	0.35*	0 (0)	1 (10)	0.32*
Phalangeal fractures	10 [7-12]	9 [7-13]	0.90	30 (47)	7 (39)	0.37*	3 (5)	0 (0)	0.47*
Finger dislocations	11 [8-13]	17 [17-17]	0.11	12 (57)	0 (0)	0.46*	1 (5)	0 (0)	0.96*
Mallet fingers	13 [11−]	-		0 (0)	-		0 (0)	0 (0)	-
***Adults n = 492***									
Carpal fractures	37 [26-58]	32 [28-57]	0.78	21 (35)	5 (31)	0.51*	2 (3)	1 (6)	0.51*
Metacarpal fractures	33 [25-49]	29 [24-47]	0.24	25 (36)	19 (32)	0.68	5 (7)	12 (20)	0.03*
Phalangeal fractures	34 [26-50]	39 [26-50]	0.79	58 (41)	30 (53)	0.15	10 (1)	5 (9)	0.45*
Finger dislocations	49 [29-63]	42 [31-48]	0.34	9 (31)	6 (22)	0.33*	1 (4)	1 (4)	0.74*
Mallet fractures	49 [39-53]	33 [27-53]	0.16	3 (13)	5 (50)	0.03*	4 (17)	3 (30)	0.33*

## 4 Discussion

In line with what might be expected, during the pandemic the absolute number of all hand injuries significantly decreased as a consequence of its concomitant restrictions (i.e. in this study by 55%). For children specifically, this reduction was even greater.

This study showed that in pre-pandemic year 2019, approximately 50% of all child injuries were sport-related and in adults this was approximately one third. This is comparable with the existing literature ^2^^,^^8^^,^^14^, ^15^, ^16^, ^17^, ^18^.

During the pandemic, there were several shifts in people's activities as a result of the restrictions. Most notable was a decrease in sport-related activities and organized team sports. Consequently, we found a substantial decrease in sport-related hand injuries in children to only 16% (i.e. a 37% reduction) and in adults to 20% (i.e. a 7% reduction). When comparing the specific underlying trauma mechanisms before and during the pandemic in this study, adults appear to have replaced organized sports with individual high or low-speed alternatives, which may not be suitable for children to engage in. This may partly explain the difference in reduction in trauma mechanism category ‘’sports'’ between children (37%) versus adults (7%).

The substantial decrease in sports participation among children and adults during the pandemic is supported by a population assessment on this topic conducted by NOC-NSF.^19^ Moreover, they reported a greater decrease in children compared to adults which match the results of this study.

During the pandemic, the proportion of metacarpal injuries increased relatively strong compared to other types of hand injuries, in both children and adults.

In children, metacarpal injuries are usually seen more often in males, with the highest incidence observed during the adolescent years.^20^ The female to male ratio in our pre-pandemic cohort was comparable with the literature 20, 21, 22. The female to male-ratio during the pandemic decreased substantially, i.e. from 1:4 to 1:10. This might be explained by the fact that metacarpal injuries were, during the pandemic, more often caused by physical interactions such as fights. It seems that during the pandemic males are at an even higher risk of developing metacarpal injuries compared to pre-pandemic conditions, albeit that in absolute number of patients, metacarpal injuries were reduced, like all other injuries.

In adult patients, metacarpal injuries were operated significantly more often during the pandemic, while no significant differences were observed in patient characteristics regarding gender or age. Notably, female adult patients were operated twice as often.

Previous studies already described an increased upper limb fracture rate resulting from bicycling or high speed cycling during the first pandemic lockdown, and this is in line with the findings regarding trauma mechanisms in this study, an increase from 3 to 8% in high speed sport hand injuries.^23^ This might explain why female patients, around the age of thirty, who engage in (high-speed) cycling, may be at a higher risk of experiencing more severe metacarpal injuries that necessitate operative treatment.

This particular target population may also be at risk for phalangeal fractures that necessitate operative treatment. Even though phalangeal fractures decrease the most in absolute number of injuries, it still represents the largest subgroup of adult hand injuries. No significant differences were observed in patient characteristics regarding gender, age or operation rate. However the proportion of female patients with phalangeal fractures raised from 42% to 53% and the operation rate multiplied by nine. This might have a the similar explanation as for metacarpal injuries, i.e. that female patients, around the age of thirty to forty, who engage in (high-speed) cycling, may be at a higher risk of experiencing severe phalangeal fractures that necessitate operative treatment.^23^

For mallet finger injuries, there was a notable increase in operation rate as well as proportion of female patients. There was notable decrease in the median age of patients. Based on trauma mechanisms underlying a mallet finger injury during a pandemic, it can be inferred that female patients in their mid-thirties who find themselves involved in unfriendly fights may be at an increased risk of requiring surgical intervention for mallet finger injuries.

### 4.1 Strengths and limitations

This study provides a thorough analysis of all patients with hand or finger injuries who presented to the ED of a large urban teaching hospital before and after the pandemic. The calendar weeks during which we included patients comprised the weeks during which the Dutch government restrictions were most strict. Herewith, it provides useful information regarding changes in incidence, patient characteristics, trauma mechanisms and operation rates following such strict lockdown and isolation restrictions. Limitations of this study include its retrospective nature, hence all data were gathered from electronic patient records, as well as the fairly small subgroups of patients.

In conclusion, restrictions during a pandemic lead to an absolute decrease in number of patients with hand and finger injuries, cause a shift in underlying trauma mechanisms from sport to physical interactions, result in a significantly higher probability of needing operative treatment when being an adult patient. Female patients, around the age of thirty-forty, who engage in (high-speed) cycling, may be at a higher risk of experiencing more severe metacarpal or phalangeal fractures that necessitate operative treatment. The information in this study might be helpful in case of any future restrictions of lockdown measures, to plan for effective and efficient trauma care accordingly.

## Informed consent

Informed consent was not sought for the present study because data was anonymized.

## Data sharing statement

Upon reasonable request to corresponding author.

## Contributor ship

DS and TG researched literature and conceptualized the study. DS and RV were involved in data curation, resources and project management. DS, TG, MB and MD were involved in investigation, methodology, formal analysis. Supervision was provided by MD. DS and TG wrote the first draft of the manuscript. All authors reviewed and edited the manuscript and approved the final version of the manuscript.

## Ethical statement

Ethical approval for this study was obtained by the institutional review board of our hospital (WO 22.129).

## Role of the funding source

The authors received no financial support for the research, authorship, and/or publication of this article.

## Funding

This work was conducted without direct financial support from any funding agency.

## Declaration of competing interest

None.
