# Supplementary material for: Trends and patterns in hand injuries during the a pandemic
Source: J Orthop Rep. 2026 Aug;5(4):None. doi: 10.1016/j.jorep.2026.100917 (PMC13491390; doi:10.1016/j.jorep.2026.100917)
Supplement: Supplementary file 1 [file mmc1.docx]

**Table S1. Proportional and absolute numbers of trauma mechanisms per type of injury per year in children**

| *Trauma mechanisms in children per year % (n)* |  | **Carpal** **fractures or dislocations** | **Metacarpal fractures or dislocations** | **Phalangeal** **fractures** | **Finger dislocations** | **Mallet fingers** | Total number of injuries per trauma mechanism |
| --- | --- | --- | --- | --- | --- | --- | --- |
| *Sports: Soccer* | *2019* | 55 (6) | 19 (4) | 14 (9) | 38 (8) | 50 (1) | 24 (28) |
|  | *2020* | - | - | - | - | - | - |
| *Sports: basketball* | *2019* | - | 5 (1) | 16 (10) | 19 (4) | - | 13 (15) |
|  | *2020* | - | - | 6 (1) | - | - | 3 (1) |
| *Sports: gymnastics, rugby, boxing* | *2019* | 9 (1) | 5 (1) | 11 (7) | 5 (1) | 50 (1) | 9 (11) |
|  | *2020* | 33 (1) | 10 (1) | - | - | - | 3 (1) |
| *Sports: Skateboarding/stepping* | *2019* | - | 10 (2) | 2 (1) | - | - | 3 (3) |
|  | *2020* | - | - | 11 (2) | - | - | 9 (3) |
| *Sports: Field hockey* | *2019* | - | 5 (1) | 8 (5) | - | - | 5 (6) |
|  | *2020* | - | - | - | - | - | - |
| *Injury caused by object* | *2019* | 27 (3) | 14 (3) | 27 (17) | 29 (6) | - | 24 (29) |
|  | *2020* | - | - | 33 (6) | - | - | 19 (6) |
| *Playing outside the house/ bicycling* | *2019* | 9 (1) | 10 (2) | 17 (11) | 10 (2) | - | 13 (16) |
|  | *2020* | 67 (2) | 20 (2) | 28 (5) | - | - | 29 (9) |
| *Physical: hit a wall* | *2019* | - | 24 (5) | - | - | - | 4 (5) |
|  | *2020* | - | 30 (3) | 11 (2) | - | - | 16 (5) |
| *Physical: friendly fight* | *2019* | - | 5 (1) | 3 (2) | - | - | 3 (3) |
|  | *2020* | - | 30 (3) | 11 (2) | 100 (1) | - | 19 (6) |
| *Physical: unfriendly fight* | *2019* | - | 5 (1) | 3 (2) | - | - | 3 (3) |
|  | *2020* | - | 10 (1) | - | - | - | 3 (1) |
| Total number of injuries per type of hand injury | *2019* | 9 (11) | 18 (21) | 54 (64) | 18 (21) | 2 (2) | 100 (119) |
|  | *2020* | 9 (3) | 31 (10) | 56 (18) | 3 (1) | - | 100 (32) |
