# Supplementary material for: Trends and patterns in hand injuries during the a pandemic
Source: J Orthop Rep. 2026 Aug;5(4):None. doi: 10.1016/j.jorep.2026.100917 (PMC13491390; doi:10.1016/j.jorep.2026.100917)
Supplement: Supplementary file 2 [file mmc2.docx]

**Table S2: Proportional and absolute numbers of trauma mechanisms per type of injury per year in adults**

| *Trauma mechanisms in adults per year % (n)* |  | **Carpal** **fractures or dislocations** | **Metacarpal fractures or dislocations** | **Phalangeal** **fractures** | **Finger dislocations** | **Mallet fingers** | Total number of injuries per trauma mechanism |
| --- | --- | --- | --- | --- | --- | --- | --- |
| *Sports: Individual low speed* | *2019* | 3 (2) | 3 (2) | 5 (7) | 10 (3) | 13 (3) | 5 (17) |
|  | *2020* | 6 (1) | 5 (3) | 2 (1) | 11 (3) | 10 (1) | 5 (9) |
| *Sports: Individual high speed* | *2019* | 5 (3) | 3 (2) | 2 (3) | 10 (3) | - | 3 (11) |
|  | *2020* | 38 (6) | 7(4) | 7 (4) | - | - | 8 (14) |
| *Sports: soccer, football, rugby* | *2019* | 7 (4) | 3 (2) | 11 (15) | 10 (3) | 4 (1) | 8 (25) |
|  | *2020* | 6 (1) | 2 (1) | 4 (2) | 4 (1) | 10 (1) | 4 (6) |
| *Sports: (Field-)/ice-hockey* | *2019* | - | 7 (5) | 9 (12) | - | - | 5 (17) |
|  | *2020* | - | 2 (1) | (2) | - | - | 2 (3) |
| *Sports: Ball sports* | *2019* | - | - | 9 (12) | 7 (2) | 8 (2) | 5 (16) |
|  | *2020* | - | - | 2 (1) | - | - | 1 (1) |
| *Injury caused by object* | *2019* | 2 (1) | 16 (11) | 21 (30) | (2) | 29 (7) | 16 (51) |
|  | *2020* | 6 (1) | 3 (2) | 26 (15) | 22 (6) | 10 (1) | 15 (25) |
| *Falling outside: tripped/ with two-wheeler* | *2019* | 65 (39) | 21(15) | 22 (31) | 14 (4) | 4 (1) | 28 (90) |
|  | *2020* | 38 (6) | 39 (23) | 28 (16) | 30 (8) | 10 (1) | 32 (54) |
| *Falling inside: tripped/ during domestic work* | *2019* | 13 (8) | 13 (9) | 10 (14) | 31 (9) | 33 (8) | 15 (48) |
|  | *2020* | 6 (1) | 7 (4) | 16 (9) | 22 (6) | 30 (3) | 14 (23) |
| *Physical: hit a wall* | *2019* | - | 23 (16) | 1 (1) | 3 (1) | - | 6 (18) |
|  | *2020* | - | 29 (17) | - | - | - | 10 (17) |
| *Physical: friendly fight* | *2019* | 2 (1) | 3 (2) | 5 (7) | - | - | 3 (10) |
|  | *2020* | - | - | 4 (2) | 4 (1) | - | 2 (3) |
| *Physical: unfriendly fight* | *2019* | 3 (2) | 9 (6) | 6 (8) | 7 (2) | 8 (2) | 6 (20) |
|  | *2020* | - | 7 (4) | 9 (5) | 7(2) | 30 (3) | 8 (14) |
| Total per type of hand injury | *2019* | 19 (60) | 22 (70) | 43 (140) | 9 (29) | 7 (24) | 100 (323) |
|  | *2020* | 10 (16) | 35 (59) | 34 (57) | 16 (27) | 6 (10) | 100 (169) |
